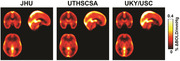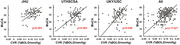# Cerebrovascular reactivity as a candidate biomarker in small vessel disease related VCID

**DOI:** 10.1002/alz.086585

**Published:** 2025-01-09

**Authors:** Hanzhang Lu

**Affiliations:** ^1^ Johns Hopkins University School of Medicine, Baltimore, MD USA

## Abstract

**Background:**

The brain’s ability to perform a cognitive task is a dynamic process and requires small blood vessels to dilate or constrict in real time to adjust blood flow in a region‐specific manner. Cerebrovascular Reactivity (CVR) measures the ability of vessels to react to vasoactive challenges. In this work, we investigated the role of CVR as a possible biomarker in small vessel disease related vascular contributions to cognitive impairment and dementia (VCID), as part of the NINDS‐funded MarkVCID study.

**Methods:**

This work consisted of 4 studies. CVR was measured by MRI signal changes in response to inhalation of 5% carbon dioxide (CO2) (Figure 1). Study 1 was a single‐site study of 72 participants (aged 69±8 years, 28 normal cognition and 44 cognitive impairment), in which we examined the relationship between CVR and cognitive function, CSF‐measured AD pathological markers, and CDR‐sum‐of‐boxes. Study 2 conducted an instrumental validation to benchmark the test‐retest reproducibility of the CVR measure. Study 3 developed an automatic, cloud‐based tool for automatic and standardized processing of CVR MRI data. Study 4 was a multi‐site biological validation study in which CVR was measured across 3 sites and data from each site were separately analyzed to investigate the relationship between CVR and cognitive function.

**Results:**

Study 1 revealed that whole‐brain CVR was associated with MoCA (β=29.64, 95% CI, 9.94 to 49.34) and global composite scores (β=4.32, 95% CI, 0.05 to 8.58), and these associations were independent of CSF‐measured amyloid and tau markers. CVR was also associated with CDR‐SB score. Study 2 revealed that inter‐scanner CoV of CVR was 6.90 ± 5.08% and had an ICC of 0.8498. Study 3 showed that CVR processing can be standardized and the processing pipeline can be accessed by any researchers around the world through an installation‐free cloud platform referred to as MRICloud (https://braingps.mricloud.org/cvr.v5). Study 4 showed that CVR was significantly associated with MoCA in data analyzed separately in each of the three sites through a pre‐defined analysis (p=0.003, 0.002, 0.046 for the three sites, respectively; when pooling data together p=3.7*10^‐6) (Figure 2).

**Conclusion:**

CVR measured with MRI is a promising candidate biomarker in VCID.